# Antagonist effects of the leek *Allium porrum* as a companion plant on aphid host plant colonization

**DOI:** 10.1038/s41598-021-83580-8

**Published:** 2021-02-17

**Authors:** Xavier Baudry, Géraldine Doury, Aude Couty, Yvelise Fourdrain, Robin van Havermaet, Marc Lateur, Arnaud Ameline

**Affiliations:** 1 UMR CNRS 7058 EDYSAN (Écologie et Dynamique des Systèmes Anthropisés), Université de Picardie Jules Verne, 33 rue St Leu, 80039 Amiens Cedex, France; 2 PCG (Provinciaal Proefcentrum Voor de Groenteteelt Oost-Vlaanderen), Karreweg 6, 9770 Kruishoutem, Belgium; 3 CRA-W (Centre Wallon de Recherches Agronomiques), Unité Biodiversité et Amélioration des Plantes, Bâtiment Emile Marchal, 4 rue de Liroux, 5030 Gembloux, Belgium

**Keywords:** Agroecology, Behavioural ecology, Ecophysiology, Ecosystem ecology, Ecosystem services, Plant ecology, Plant signalling, Chemical ecology

## Abstract

Combining a non-host plant (companion plant or CP) with a target cultivated plant is considered as a promising strategy to reduce pest pressure. Among the companion plants (CP) commonly used in integrated systems, those belonging to the *Amaryllidaceae* family (chives, garlic, onion, leek) exhibit characteristics related to certain volatile organic compounds (VOCs) with promising repellent potentialities. The aim of this work was to investigate the potential disruption of sweet pepper (host plant) colonization by the green peach aphid (*Myzus persicae*) when exposed to leek (*Allium porrum*) as a CP. Retention/dispersion, EPG and clip-cage/Petri dish laboratory experiments were thus performed to study the effect of leek VOCs on aphid settlement/migration, feeding behavior and life history traits parameters, respectively. This work revealed that leek as a CP had a negative effect on aphid feeding behavior, by disturbing the balance between phloem and xylem sap ingestion, but had no influence concerning aphid settlement. Surprisingly, leek as a CP triggered some unexpected probiotic effects on certain life history traits such as aphid survival, biomass, and fecundity, suggesting a possible hormetic effect of leek VOCs on aphid physiology. The possibility of experience-induced preference of aphids for leek VOCs was also discussed.

## Introduction

Phytophagous insects evolve in complex and diverse environments and have to deal with various information sources, including odors, in order to achieve successful host plant selection and their subsequent reproductive cycle. Plants associations have long been used in a context of integrated protection of crops to sustainably disturb the interactions between insects and their host plants^1^. Combining a non-host plant (companion plant or CP) with a target cultivated plant is considered as a promising strategy to reduce pest pressure. CPs can act in various ways, depending on their physical and/or chemical characteristics (for review see Parker et al., 2013). One of the main benefits of CPs lies with their propensity to emit volatile organic compounds (VOCs) that can contribute to the protection of the target plant from pests by mechanisms involving chemically masking properties^3,4^ and/or repelling effects^5^.

Aphids are one of the world’s major insect pest groups causing serious economic impacts in a wide range of agrosystems. The use of CPs can represent an effective strategy to disturb their host plant colonization process^6^ at various steps comprising adult migration, landing, settlement and reproduction^7,8^. The emission of volatile organic compounds (VOCs) by CPs has been shown to significantly modify host plant location by aphids by repelling them from their host plant^9^ and/or by reducing the host plant attractivity by masking its olfactory signature^10^. Several works have demonstrated that using CPs can also impact aphids life history traits and significantly reduce fecundity^11^. However, to our knowledge, the potential disturbance capacity of CPs on the crucial step of aphid probing and feeding behavior leading to plant acceptance remains largely unknown.

Most studies that have been performed on CPs commonly known as “odoriferous plant species”^12^, particularly from the *Lamiaceae*^13,14^, *Asteraceae*^15–17^, and *Apiaceae* botanical families^18,19^ have shown numerous examples of efficient association. However, some studies also report the relative failure of some plant volatiles combinations that could be explained by the fact that insects experience of CP VOCs may modify or even nullify their innate preference for specific olfactory cues^20^. Indeed a process of plant odor learning by juveniles may influence adult behavior through imprinting (sensitization or habituation) or associative learning^21^. The *Amaryllidaceae* family plants also exhibit interesting features that could make them promising CPs candidates^22,23^. Indeed, species belonging to the *Allium* genus have been identified as significant sources of VOCs impacting insect pests colonization^24,25^. The success of *Allium spp.* VOCs against pests is generally attributed to the presence of different disulfide compounds such as dimethyl disulfide (DMDS) released following herbivory or any mechanical lesion^26^. On aphids,^27^ revealed the masking and repellent properties of leek (*Allium porrum* L.) VOCs towards *Myzus persicae* (Sulzer). Field bioassays intercropping wheat (*Triticum aestivum* L.) with garlic (*Allium sativum* L.) showed a lower infestation by *Sitobion avenae* (Fabricius) on wheat associated with garlic in comparison to wheat monoculture, and better yields due to this association^28^.

This study explores how leek could be used as a CP to impact aphid colonization process. The green peach aphid (*M. persicae*) was used as a pest model and sweet pepper (*Capsicum annuum* L.) as the target host plant. The potential disturbance of leek VOCs was evaluated on (1) aphid orientation behavior using a dispersion/retention test, (2) aphid feeding behavior using the electropenetrography technic (EPG) and (3) aphid performance using clip-cages and Petri dishes tests. The potential effect of aphids imprinting was also evaluated through their nymphal pre-exposition to leek volatiles.

## Materials and methods

### Plants and insect material

All the experiments were performed using sweet pepper (*Capsicum annuum L.* cv. ‘Sprinter’) as the target plant associated with leek (*Allium porrum L*. cv. ‘Oslo’) as the companion plant. Plants were provided as seeds by the “Provinciaal Proefcentrum voor de Groenteteelt Oost-Vlaanderen” (PCG, Belgium). Sweet peppers and leeks were grown in separate rooms to limit the impregnation of sweet peppers by leek VOCs, respectively in 7 × 7 × 6 cm and 9 × 9 × 10 cm pots containing potting soil (NPK 18–10-20, 0.5 kg/m3, FLORAGARD) under controlled conditions (24 ± 1 °C, 60 ± 5% relative humidity (RH), and 16L:8D photoperiod at 2,5 klux.). For all the experiments, sweet peppers and leeks were used five weeks and 12 weeks after sowing, respectively.

The *Myzus persicae* (Sulzer) (Hemiptera: Aphididae) colony was established from one parthenogenetic aphid collected in 1999 in a potato field near Loos-en-Gohelle (France) and maintained on sweet pepper plants (*Capsicum annuum* var. ‘Sprinter’) placed in 50 × 50 × 50 cm ventilated plastic cages under the same conditions as described above. All the experiments were performed using young apterous adults (9 ± 2 day-old, corresponding to their pre-reproductive period) synchronized on sweet pepper leaves embedded in 1.5% agar in Petri dishes (Ø 90 mm).

In order to assess the influence of leek used as a companion plant, behavior and physiology of aphids on the target plant alone “P” (sweet pepper, either a potted plant or a leaf embedded in Agar in a Petri dish) were compared to those on the target plant associated to leek “PL” (leek was presented either as a potted plant 5 cm apart from the sweet pepper plant, or as cut leaves deposited next to the sweet pepper leaf in a Petri dish). At the beginning of each experiment and then every 24 h, leek leaves were cut at their terminal part in order to stimulate the emission of VOCs. All experiments (unless specified otherwise) were performed using naive aphids, i. e. that had never been in contact with leek leaves and/or submitted to leek VOCs.

### Aphid settlement/migration

A preliminary choice test was performed to evaluate the putative repulsive properties of leek and the putative attractivity of sweet pepper. An apterous aphid was placed in the middle of an 8 cm × 2 cm plastic bridge linking a pot containing a sweet pepper plant (P) to another pot containing a leek plant (PL). Each individual aphid was then observed for 5 min after being released and the time before the aphid first contact with either sweet pepper plants from the two conditions (P or PL), and the pepper plant receiving this first contact were recorded. The experiment was repeated on 36 different aphids.

Aphid emigration and settlement preference were tested in chambers allowing contact-based, odor-based and visual cues. The experimental set-up used was adapted from^29^. In these bioassays, we assessed the propensity of apterous aphids to emigrate from a sweet pepper that was associated, or not, with leek. Ten aphids were released onto leaves of sweet pepper (P) or sweet pepper associated with leek (PL) (the “release” plant) coupled with a second sweet pepper plant (the “choice” plant), with the opposite status (PL or P). The two plants in the set-up were linked by a bridge allowing aphids to move between sweet pepper plants. The whole set-up was placed in an aerated room where the “release” and the “choice” plants locations were alternated to avoid any environment variation between each condition. Aphids were then counted on each plant 24 h after deposition. To minimize external stimuli, the cage was positioned within four white foam cardboard walls and a white fluorescent tube was positioned above. Each test was repeated 20 times.

### Aphid feeding behavior

The feeding behavior of *M. persicae* was studied using the electropenetrography technique^30,31^ by comparing the feeding behavior of aphids placed onto a sweet pepper alone (P) with that of aphids placed onto a sweet pepper associated with a leek (PL). Eight hours recordings were carried out in the middle of the 16 h of photophase under controlled conditions (24 ± 1 °C, 60 ± 5% RH and 16L:8D photoperiod, 2,5 klux). The records were obtained according to a set-up consisting in sticking a thin gold wire (Ø 20 μm and 2 cm long) with a conductive silver glue (EPG systems, Wageningen, the Netherlands) on the dorsal part of the aphid’s abdomen, which was connected to an electrical closed circuit comprising the aphid and its sweet pepper host plant. An eight channels set-up allowed a simultaneous acquisition of the feeding behavior of eight aphids. Acquisition and analysis of the EPG waveforms were carried out with EPG Stylet + software (EPG Systems, www.epgsystems.eu). Relevant aphid behavior EPG parameters were calculated with EPG-Calc 6.1.7 software^32^. The feeding behavior of 29 aphids for the P condition and 26 aphids for the PL condition was recorded. Aphids were placed on the abaxial part of top two fully expanded leaves.

Eight different EPG parameters were used to assess the feeding behavior of *M. persicae* with or without associated leek. The total duration of probing (s_Pr) and the number or probes (n_Pr) corresponding to the general probing behavior of the aphid in the leaf were evaluated. We also evaluated the total duration of pathway phase (s_C) corresponding to the insertion of aphid’s stylets in mesophyll tissues, the total duration of phloem salivation (s_E1) and the time to first phloem phase (t_1stE2), along with the total duration of sustained phloem phase (s_SE2) respectively corresponding to the time before the first ingestion of phloem sap and the duration of phloem sap ingestion that was longer than 15 min. Finally, the total duration of ingestion of xylem sap (s_G) and the total duration of derailed stylet phase (s_F) were also calculated.

As in the study assessing aphid life history traits aphids in the PL condition were continuously reared in the presence of leek VOCs, an additional bioassay was performed. The aim was to evaluate the possibility of behavioral habituation or experience-induced preference in those aphids. To this end, six day-old aphid nymphs were synchronized in Petri dishes as described previously before being transferred in a new Petri dish containing a sweet pepper leaf embedded in agar with 3 g of cut leek leaves deposited on the substrate that were then changed every 24 h during the following three days. The feeding behavior of 22 aphids (pre-exposed, pPL, versus naive, PL) was studied on sweet pepper associated with leek using EPG under the same conditions as described above.

### Aphid physiology

Physiological and biomass studies were performed using “clip-cages” adapted from^33^. Each clip-cage was composed of a plier supporting an acrylic transparent cylinder (Ø 15 mm, 8 mm long) closed by a nylon gauze while the airtightness on the leaf was secured thanks to felt. Clip-cages were placed on the abaxial part of top two fully expanded leaves of sweet pepper alone (P) or sweet pepper associated with leek (PL). Physiological and biomass studies were also performed in Petri dishes using aphids synchronized as described previously. A sweet pepper leaf was embedded in 1,5% agar with 3 g of cut leek leaf for the PL condition and without leek for the P condition. All the experiments described below were carried out both in clip-cages and in Petri dishes under controlled conditions (24 ± 1 °C, 60 ± 5% RH and 16L:8D photoperiod, 2,5 klux).

Concerning physiological studies, a single 9 ± 2 day-old aphid was placed per leaf. Then, individual survival and fecundity were noted every day for each condition for nine days, a duration equivalent to that of the pre-reproductive period. A total of 28 (P) and 30 (PL) individuals for clip-cage experiments and a total of 23 (P) and 23 (PL) aphids for Petri dish experiments were respectively used to evaluate the survival rate and the daily fecundity, while a total of 22 (P) and 21 (PL) aphids for clip-cage experiments and a total of 13 (P) and 21 (PL) aphids for Petri dish experiments were used to evaluate the total fecundity in each condition.

Individual biomass was measured using groups of synchronized first-instar nymphs (less than 24 h old) of *M. persicae* which were obtained from parthenogenetic adult females placed on sweet pepper leaves. After 24 h, the adult females were removed from the leaf while the newly larviposited *M. persicae* were kept on sweet pepper leaf for the 8 following days. To test the effects of leek as a companion plant on aphid biomass, 9 days-old aphids were randomly selected, frozen at -80 °C in individual tubes and subsequently weighed one at a time, using a precision electronic scale (Mettler M3, class 1, Max: 3 g, Low: 1 µg, T =  − 3G [dd] = 1 µg). A total of 27 (P) and 25 (PL) individuals for clip-cage experiments and 19 (P) and 26 (PL) individuals for Petri dish experiments were weighed.

### Statistical analysis

Statistical analyses of data concerning the retention/dispersion bioassays, EPG tests, and biomass and total fecundity measures were performed using a non-parametric Mann–Whitney U-test for independent samples as they did not follow a normal distribution (Shapiro test) and homoscedasticity (Fisher test) was not respected. Choice test responses were analyzed using a Chi-square test. Daily fecundity data were analyzed using a general linear model (GLM) using a Poisson distribution. When a significant effect of one of the main factors was detected or when an interaction between factors was significant, a pairwise comparison using least-squares means (package R: “lsmeans”) (p-value adjustment with Tukey method) was used at the 0.05 significance level to test for differences between treatments. Survival was modelled using the Cox proportional hazards (CPH) model and cases where the given event did not occur were treated as censored. The assumption of validity of proportional hazards was checked using the functions “coxph” and “cox.zph” (package R: “survival”). When a significant effect of one of the main factors was detected or when an interaction between factors was significant, a pairwise comparison using Estimated Marginal means (package R: “emmeans”) (p-value adjustment with Tukey method) was used at the 0.05 significance level to test for differences between treatments. R software version 3.6.2^34^ was used for all statistical analyses.

### Ethical approval

The article does not contain any studies with human participants or vertebrate animals.

## Results

### Aphid host plant preference

The preliminary choice experiment between a sweet pepper or a leek plant revealed that aphids preferentially migrated and settled on sweet pepper (Fig. S1) (Chi squared test, χ^2^ = 7.11, p = 0.0078).

**Figure 1:**
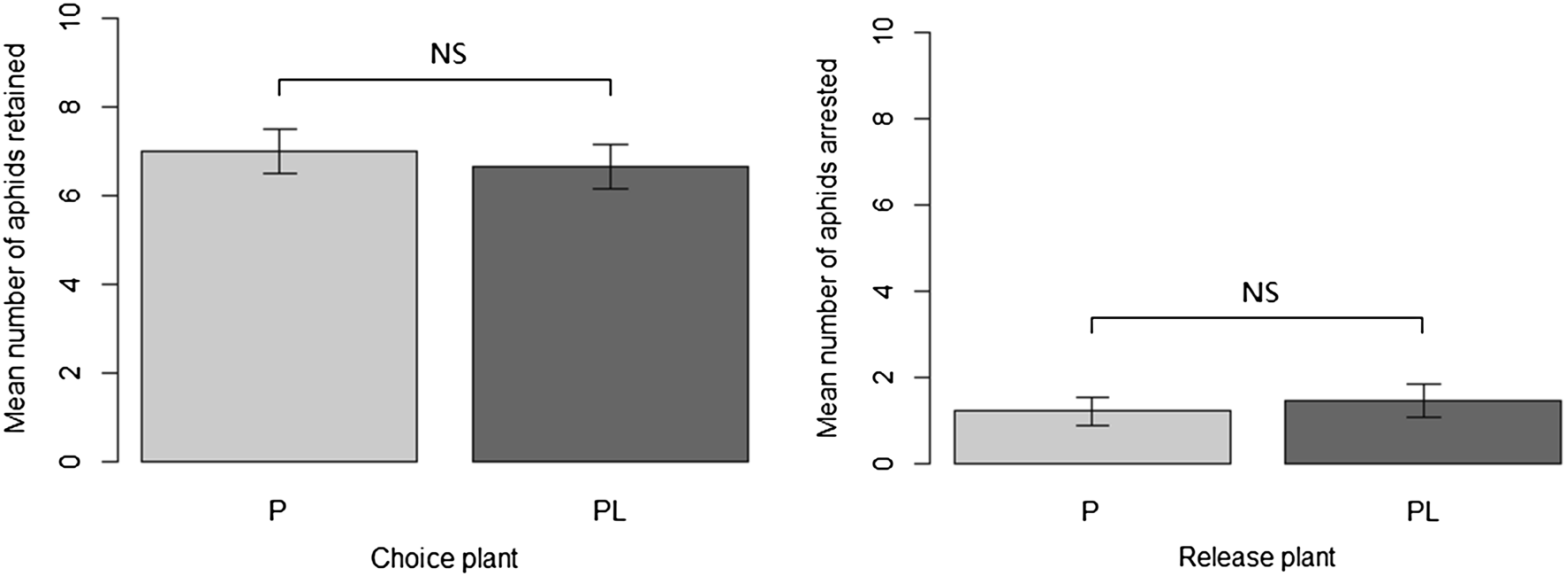
Behavioral responses (mean ± SEM) of *M. persicae* to contact, volatile and visual cues of sweet pepper alone (P) and sweet pepper associated with leak (PL) after 24 h. Ten aphids were allowed to disperse from leaves of a sweet pepper to an adjacent “choice plant” of the opposite status. Twenty replicates were performed for each condition. NS indicate a non-significant difference between sweet pepper and sweet pepper associated with leek (Mann–Whitney U-test).

Leek companioning had no significant effect on aphid settlement-migration between a sweet pepper alone and a sweet pepper associated with leek (Fig. 1). Sweet pepper as a release plant retained aphids in a similar way, whether associated or not with leek (Mann–Whitney U-Test ; U = 220, p = 0.5942). Similarly, aphids arrested similarly on sweet pepper as a choice plant, whether associated or not with leek (Mann–Whitney U-Test ; U = 187.5, p = 0.7332).

### Aphid feeding behavior

EPG study revealed significant effects of leek volatiles on several feeding behavior parameters. The time to first phloem ingestion (t > 1E2) was significantly longer on PL (by ca. one hour) (Mann–Whitney U-test, P vs PL: p < 0.05). In addition, the total duration of sustained phloem sap ingestion (s_SE2) was severely modified on PL (Mann–Whitney U-test, P vs PL: p < 0.05) as it was significantly reduced by half compared to that on P. Finally, the association of leek with sweet pepper also had a significant effect on the total duration of xylem phase, that was increased by 40% compared to that on sweet pepper alone (Mann–Whitney U-test, P vs PL: p < 0.01). The total duration of stylets presence in plant tissues (s_Pr) was not significantly affected by the association of leek with sweet pepper (Mann–Whitney U-test, P vs PL: p > 0.05) and represented 90% of the total recording time (Table 1). There was no significant effect of the association of leek with sweet pepper (Mann–Whitney U-test, P vs PL: p > 0.05) on the duration of stylets activity (s_C) within mesophyll, on the duration of salivation activity (s_E1) within phloem and on the duration of the stylet derailment (s_F).

**Table 1:** Feeding behavior parameters (mean + /- SEM) of *M. persicae* on sweet pepper depending on the plant status (sweet pepper associated or not with leek) and on the aphid status (pre-exposed or not to leek VOCs). Behavior of naive *M. persicae* on sweet pepper alone (P) was compared to that of naive *M. persicae* on sweet pepper associated with leek (PL) and then behavior of naive *M. persicae* on sweet pepper associated with leek (PL) was compared to that of pre-exposed *M. persicae* to leek VOCs on sweet pepper associated with leek (pPL).

	Naive aphids on sweet pepper alone (P)		Naive aphids on sweet pepper associated with leek (PL)		Preexposed aphids on sweet pepper associated with leek (pPL)		P vs PL		PL vs pPL	
	n		n		n		U	p-value	U	p-value
**General probing phase (Pr)**										
Total duration of probing (min. s_Pr)	29	437.10 ± 6.12	26	434.11 ± 4.64	22	427.78 ± 6.50	429	0.388	270	0.751
Number of probes (n_PR)	29	14.17 ± 1.84	26	14.54 ± 1.99	22	13.04 ± 1.10	366.5	0.866	298	0.812
Time to first probe (t > IPr)	29	6.77 ± 1.10	26	10.39 ± 2.63	22	4.55 ± 0.65	384	0.914	319	0.334
**Pathway phase (C)**										
Total duration of pathway phase (min. s_C)	29	156.05 ± 16.55	26	181.82 ± 11.74	22	133.01 ± 7.61	299	0.193	421	**0.005**
**Pholem phase (E)**										
Total duration of salivation phase (min. s_E1)	29	43.91 ± 7.55	22	43.72 ± 7.17	18	36.30 ± 6.53	295	0.658	229	0.411
Time to first phloem phase (min. t > 1E2)	17	120.82 ± 27.78	14	193.27 ± 27.89	10	236.36 ± 21.78	173	**0.032**	54	0.371
Duration of sustained phloem phase (min. s_sE2)	17	239.00 ± 38.83	10	120.53 ± 32.23	10	136.81 ± 21.11	41	**0.027**	45	0.739
**Other phases**										
Total duration xylem phase (min. s_G)	27	57.00 ± 11.04	26	97.55 ± 10.97	21	102.11 ± 13.88	197	**0.006**	290	0.727
Total duration of derailed stylet phase (min. s_F)	10	119.69 ± 25.67	14	122.25 ± 26.16	19	122.35 ± 9.80	74	0.841	119	0.628

P values < 0.05 (Mann–Whitney U-test) are written in bold; n: sample size.

Concerning the putative effect of aphids nymphal pre-exposition to leek VOCs, the only significant impact of aphids nymphal pre-exposition to leek consisted in a shorter duration of stylets activity in mesophyll (Mann–Whitney U-test, PL vs pPL: p < 0.01). However there was no significant difference between naive (PL) and pre-exposed (pPL) aphids on sweet pepper associated with leek in terms of total duration of general stylets activity within plant tissues (s_Pr) (Mann–Whitney U-test, PL vs pPL: p > 0.05), within phloem (Mann–Whitney U-test, t > 1E2, PL vs pPL: p > 0.05 and s_SE2, PL vs pPL: p > 0.05) and within xylem (Mann–Whitney U-test, PL vs pPL: p > 0.05).

### Aphid performance

**Figure 2:**
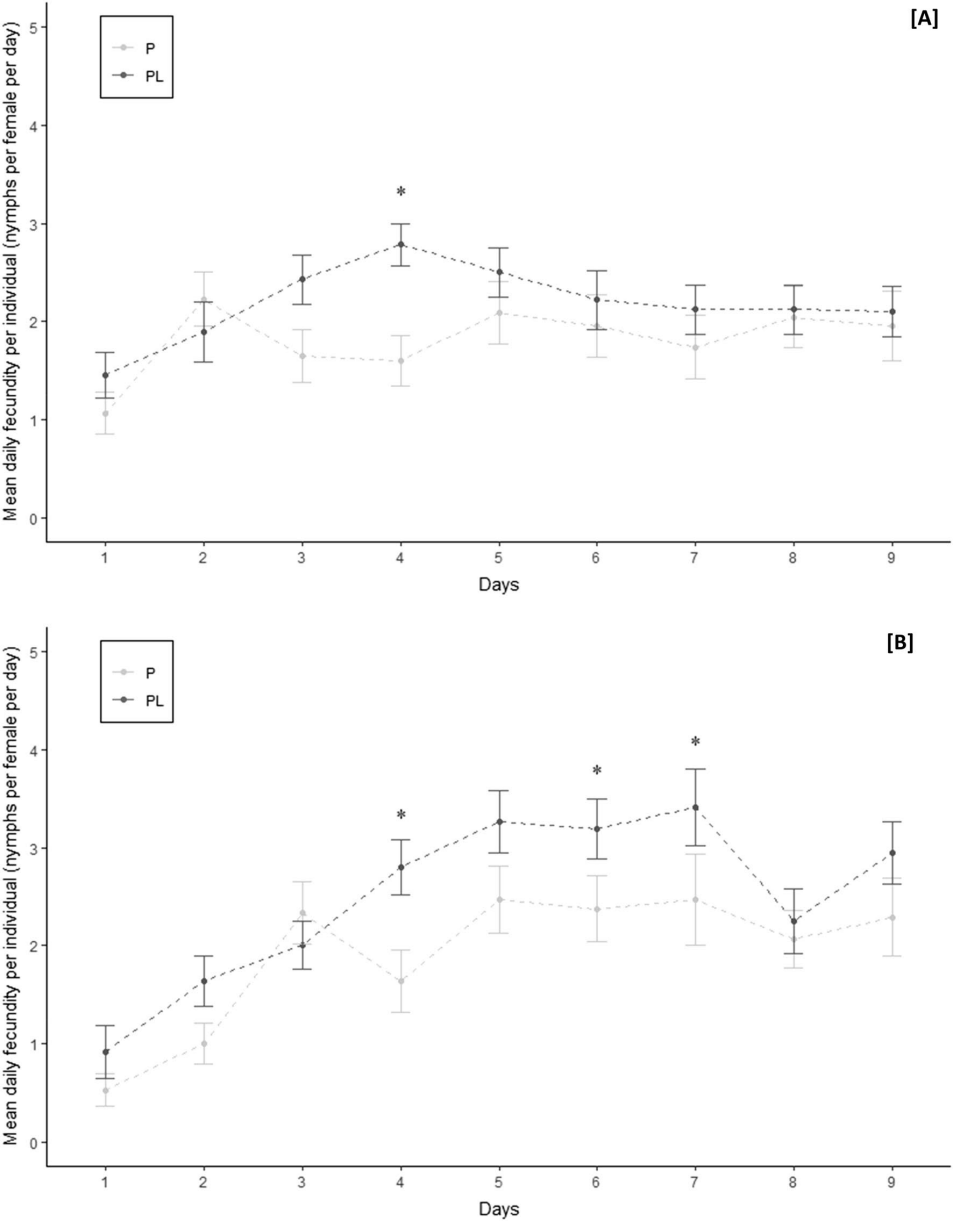
Mean daily fecundities (+ /- SEM) of *M. persicae* during the nine days following their deposition on sweet pepper alone (P) or sweet pepper associated with leek (PL). (**A**) in clip-cage (**B**) in Petri dish. (mean ± SEM). *: p < 0.05 (GLM).

In clip-cages, the total fecundity measured at nine days was 20% greater for aphids reared on sweet pepper associated with leek (Mann–Whitney U-test, clip cage, p < 0.05). Regarding the measures of daily fecundity, the number of larvae produced at four days was twice greater on PL in comparison to P (GLM, clip-cage, p < 0.01) while there were no differences for the other days (Fig. 2A). There was no significant difference in terms of survival rate or biomass between aphids reared on sweet pepper alone or associated with leek (Table 2) (Mann–Whitney U-test, clip-cage, p > 0.05).

**Table 2:** Comparisons of fitness parameters (means + /- SEM) of *M. persicae* reared on sweet pepper with (PL) or without (P) associated leek in clip-cage and in Petri dish.

	Sweet pepper (P)		Sweet pepper associated with leek (PL)		U/χ^2^	p-value
	n		n			
**Clip-cage**						
Survival rate	28	0.68	30	0.69	χ^2^ = 0.37	0.542
Total fecundity	22	17.15 ± 1.39	30	21.58 ± 1.22	U = 66	**0.036**
Adult biomass (µg)	27	271.44 ± 38.44	25	267.40 ± 12.84	U = 277.5	0.276
**Petri dish**						
Survival rate	23	0.65	23	0.91	χ^2^ = 4.56	**0.033**
Total fecundity	13	19.38 ± 0.33	21	24.95 ± 1.84	U = 285.5	**0.050**
Adult biomass (µg)	19	205.26 ± 17.96	26	256.92 ± 12.87	U = 145.5	**0.020**

P values < 0.05 (Mann–Whitney U-test and Cox Model) are written in bold; n: sample size; χ^2^: Chi square.

In Petri dishes, aphids survival rate was significantly smaller when reared on sweet peppers alone (P) compared to sweet peppers associated with leek (PL) (Cox model, Petri dish, p < 0.05) with a 65% survival rate against 91%, respectively. The total fecundity was also significantly greater on PL condition (Mann–Whitney U-test, Petri dish, p ≤ 0.05). In addition, the daily fecundity of aphids was significantly greater at four, six and seven days after being placed on PL condition (Fig. 2B) (GLM, d4: p < 0.01 ; d6: p < 0.02 ; d7: p < 0.05). Finally, when aphids were reared on PL, their biomass was 20% significantly greater than that of aphids on P (Mann–Whitney U-test, p < 0.05).

## Discussion

This study revealed contrasting effects of leek as a companion plant on aphid colonization. While leek as a CP had a negative effect on aphids feeding behavior, no effect was observed concerning their orientation behavior. Surprisingly, leek as a CP triggered some unexpected positive effects on certain physiological parameters such as aphid survival, biomass and fecundity.

Our preliminary choice experiments opposing a single sweet pepper plant to a single leek plant indicated that *M. persicae* preferred to orientate and settle on sweet pepper rather than on leek. However, settlement-migration tests revealed that aphid orientation behavior was not impacted by the presence of leek as a CP. Aphids were equally retained on sweet pepper release plants, regardless of sweet pepper association with leek. They were also equally likely to settle on sweet pepper choice plants. This is in accordance with the work of^27^ showing that although *M. persicae* was attracted by sweet pepper and repelled by chive VOCs (*Allium schoenoprasum L*.), the association of those odors was neither repellent or attractive on *M. persicae*, a result that could be due to a masking or disrupting action of the CP on the host plant attractivity.

Among the main VOCs of *Allium spp*., the emission of dimethyl disulfide (DMDS) and dimethyl trisulfide (DMTS) as well as diallyl disulfide (DADS) and diallyl trisulfide (DATS) released by garlic leaves have been shown to be significantly stimulated after mechanical lesions^25,35,36^. The insect repellency properties of these compounds were particularly demonstrated with DMDS^24^. However, even though in our study leeks were mechanically damaged to stimulate the emission of some *Allium* VOCs, it cannot completely be ruled out that their concentration could have been too low to modulate host plant location, or that other mechanisms could be involved such as a masking and/or disruption of the sweet pepper attractivity.

The presence of leek as a CP induced a disruption of the feeding behavior of *M. persicae* on sweet pepper. Although the total time allocated to stylet penetration (i.e. general probing phase) was not affected, the relative balance between the time spent to ingest phloem sap (s_sE2) and the time dedicated to xylem sap ingestion (s_G) was altered. Leek as a CP did not seem to impact any of the behavioral phases occurring before the initiation of sap ingestion. Not only the time to first probe (t > 1Pr), but also the parameters of the pathway phase were unchanged, suggesting that the modifications of *M. persicae* feeding behavior in the presence of leek were probably not due to physical or chemical changes in epiderm and/or mesophyll tissues. Indeed the hypothesis that leek VOCs could be adsorbed in the sweet pepper leaf cuticle can be ruled out here, although such phenomena have already been shown in other studies^37,38^ and particularly in a study involving *Allium spp*. VOCs^39^. It should also be noted that the presence of leek did not induce greater numbers of probes (n_Pr) performed by *M. persicae*, thus suggesting a non-repellent effect of leek VOCs.^40^ showed that the repellent effect of *Tagetes patula* (marigold) or *Ocimum basilicum* (basil) translated into greater numbers of probes (n_Pr) and a shorter total duration of probing (s_Pr) performed by *M. persicae* on sweet pepper.

The modifications of *M. persicae* feeding behavior observed in our study in the presence of leek are more likely to be attributed to changes occurring in non-superficial tissues of the host plant, such as those affecting vascular tissues. Indeed, the duration of sweet pepper sustained phloem sap ingestion was significantly shorter while the time to first phloem sap ingestion was longer. The perturbation of *M. persicae* sustained phloem sap ingestion when leek was present could globally reflect their host plant acceptance disturbance^8^. This is also in accordance with the study of^41^, who worked on *M. persicae* feeding behavior on *Tanacetum vulgare* sprayed with *A. cepa* extracts and showed a post-ingestional deterrent activity previously defined by^42^. Our study also demonstrated that leek as a CP enhanced sweet pepper xylem sap ingestion by aphids. A longer duration of xylem sap ingestion is generally considered as an aphid stress indicator^43,44^. In our case, this stress could be attributed to a negative impact of leek on sweet pepper palatability. It cannot be completely ruled out that volatiles released by the nearby leek plant could have directly enhanced induced sweet pepper defense mechanisms toward *M. persicae*. Certain VOCs have been shown to stimulate neighboring plants to adjust their defenses at the right time and subsequently reduce herbivore feeding damages^45–47^.

The physiological studies were carried out both in clip-cages and in Petri dishes. Clip-cages bioassays ensured experimental conditions similar to those used for the EPG tests whereas Petri dish bioassays are routinely used in ecotoxicology studies^40^. The physiological studies revealed an unexpected probiotic effect of leek on *M. persicae*. When aphids were submitted to VOCs delivered by freshly mechanically cut leek leaves in clip-cages, their daily fecundity was surprisingly positively impacted. Interestingly, the observed positive effects were exacerbated when such VOCs were emitted by freshly cut fragments of leek leaves enclosed in a Petri dish with aphids, as their physiological parameters (survival, fecundity and biomass) were all positively impacted. These results appear to be in contradiction with most works studying the effect of non-host plant VOCs on aphid physiology. Previous studies where insects inhaled essential oils VOCs showed intoxication leading to greater mortality^48–51,51^. Other studies indicated a negative impact of VOCs from non-host plants on insects fecundity^52–54^, although we are not aware of the description of such effects following exposure to leek VOCs. Our study revealed that aphid fecundity, survival, and biomass were all globally greater, particularly in the presence of leek in Petri dishes set-ups. To our knowledge, this is the first time that such a phenomenon is observed for aphids, even though *Allium spp.* VOCs have been demonstrated to be able to positively influence the fecundity and egg hatchability of *Bombyx mori* females that had been exposed to those odors during their larval development^55^. Our results seem to be due to a probiotic effect of leek VOCs that could be explained by hormesis as already described in aphids by^56^. Hormesis is a biological phenomenon where exposure to high stressors, such as pesticides, is inhibitory, whereas low doses are stimulatory^57^. Such a situation was reported with sublethal doses of Imidaclopride, an insecticide, that could improve *M. persicae* fecundity even if this substance is highly toxic at higher doses^58,59^. In our case it is possible that leek VOCs, so far known for their repellent properties, were delivered here in a concentration that stressed aphids during their feeding behavior (as shown by a greater xylem sap consumption), which ultimately resulted in a stimulation of their fecundity. This is all the more striking as leek VOCs negatively impacted phloem sap ingestion by aphids. Nonetheless, it should be noted that aphids were naive to leek odors, to which they were only punctually exposed during the 8 h of EPG bioassays, whereas aphids tested in the physiology bioassays were exposed to leek VOCS sources that were renewed every 24 h for at least 9 days. Such a constant and continuous exposure of aphids to leek VOCs throughout the duration of bioassays could have possibly resulted in an habituation or experience-induced preference, as described by Stephens^60^. According to this author, experience-induced preference for repellents would allow insects to retain a certain flexibility when dealing with uncertainty in their environment, that can lead them to oviposit on poorly suitable host plants when highly suitable plants are not available. In order to test for the existence of habituation and/or experience-induced preference, we also assessed the feeding behavior of aphids that had been pre-exposed to leek VOCs during their nymphal development. However, our results showed that phloem sap ingestion remained as low as when aphids had not been pre-exposed to leek VOCs, thus invalidating the involvement of any habituation or experience-induced preference. Moreover, the fact that pre-exposed aphids kept ingesting xylem for long periods of time indicates they were also stressed, thus comforting the involvement of an hormetic effect.

## Conclusion

To conclude, the effects observed here on aphid behavior and physiology in laboratory bioassays suggest that the use of leek as a companion plant may not be suitable in a context of pest management on sweet pepper. Indeed, although leek VOCs induced an alteration of the feeding behavior of *M. persicae*, they also positively impacted physiological parameters suggesting that aphid populations may build-up on sweet pepper crops even when leek is companioning. It is also possible that the way VOCs were delivered here was not sufficiently efficient. Using leek essential oils delivered by an appropriate dispenser device could ensure the diffusion of higher concentrations of the VOCs in the environment, as demonstrated with *Allium sativum* and *Allium tuberosum* essential oils inducing negative effects on Hemiptera survival^61,62^. This would also enable to explore the quantitative effects of leek VOCs, as the effects of aphid exposure to low doses of leek essential oils might help confirming hormesis while high doses should result in toxicity. Finally, measuring the potential transgenerational effect of leek companioning would be worth investigating.

## Supplementary Information

Supplementary Figure.
