# Supplementary material for: Antagonist effects of the leek Allium porrum as a companion plant on aphid host plant colonization
Source: Sci Rep. 2021 Feb 17;11:4032. doi: 10.1038/s41598-021-83580-8 (PMC7889937; doi:10.1038/s41598-021-83580-8)
Supplement: Supplementary file 1 — Supplementary Figure. [file 41598_2021_83580_MOESM1_ESM.pdf]

Title: Antagonist effects of the leek *Allium porrum* as a companion plant on aphid host plant colonization

Baudry Xavier[1], Doury Géraldine[1], Couty Aude[1], Fourdrain Yvelise[1], van Havermaet Robin[2], Lateur Marc[3], Ameline Arnaud[1*]

1. UMR CNRS 7058 EDYSAN (Écologie et Dynamique des Systèmes Anthropisés), Université de Picardie Jules Verne, 33 rue St Leu, F-80039 Amiens Cedex, France

2. PCG (Provinciaal Proefcentrum voor de Groenteteelt Oost-Vlaanderen), Karreweg 6 – 9770 Kruishoutem, België

3. CRA-W (Centre Wallon de Recherches Agronomiques), Unité Biodiversité et Amélioration des Plantes, Bâtiment Emile Marchal, 4 rue de Liroux, 5030 Gembloux, Belgique

* Correspondence: Tel.: +33 3 22 82 75 56; Fax: +33 3 22 82 75 47

E-mail address: arnaud.ameline@u-picardie.fr

Supplementary materials

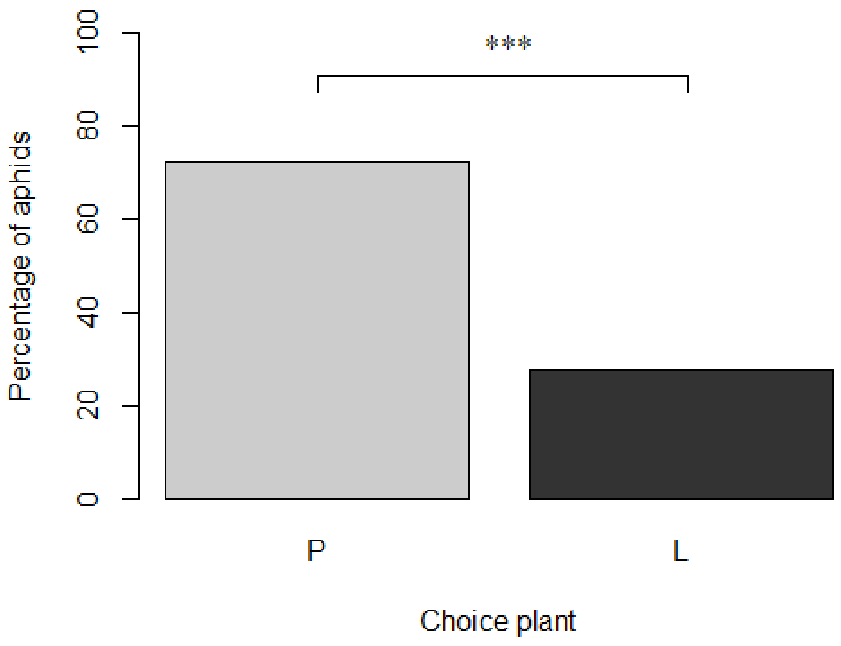

Figure S1. Percentage of aphids settling on sweet pepper (P) or leek (L) when given a choice between the two plants. Thirty-six individual releases of aphids were performed. *** indicate a significant difference (P<0.001, Chi squared test).
